# Ultrafast Demagnetization Control in Magnetophotonic Surface Crystals

**DOI:** 10.1021/acs.nanolett.2c00769

**Published:** 2022-11-02

**Authors:** Kshiti Mishra, Richard M. Rowan-Robinson, Agne Ciuciulkaite, Carl S. Davies, Alexandre Dmitriev, Vassilios Kapaklis, Alexey V. Kimel, Andrei Kirilyuk

**Affiliations:** † Radboud University, Institute for Molecules and Materials, Heyendaalseweg 135, 6525 AJNijmegen, The Netherlands; ‡ Department of Physics and Astronomy, Uppsala University, Box 516, SE-75120Uppsala, Sweden; § FELIX Laboratory, Radboud University, Toernooiveld 7, 6525 EDNijmegen, The Netherlands; ∥ Department of Physics, University of Gothenburg, SE-412 96Göteborg, Sweden

**Keywords:** magnetoplasmonics, magnetophotonics, ultrafast magnetization dynamics, all-optical switching, demagnetization, surface lattice resonances

## Abstract

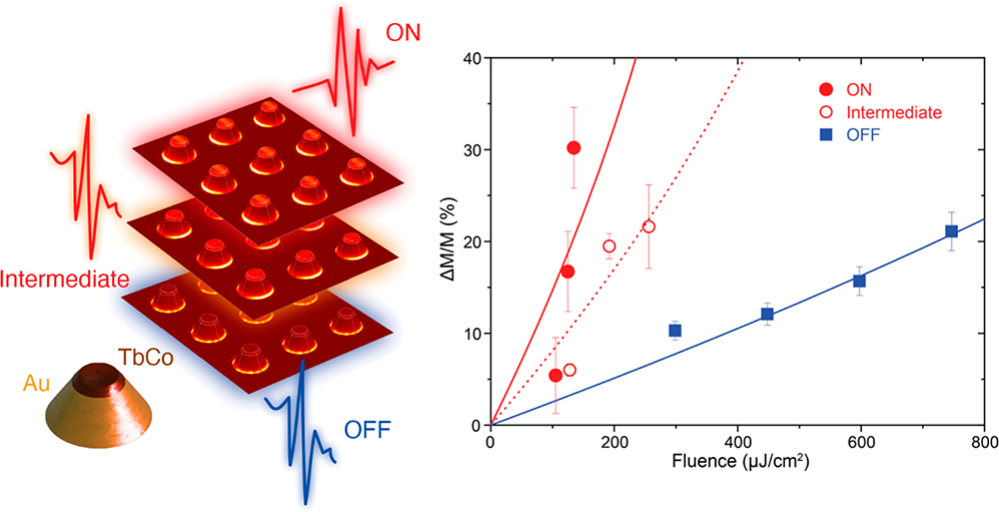

Magnetic memory combining plasmonics and magnetism is poised to dramatically increase the bit density and energy efficiency of light-assisted ultrafast magnetic storage, thanks to nanoplasmon-driven enhancement and confinement of light. Here we devise a new path for that, simultaneously enabling light-driven bit downscaling, reduction of the required energy for magnetic memory writing, and a subtle control over the degree of demagnetization in a magnetophotonic surface crystal. It features a regular array of truncated-nanocone-shaped Au-TbCo antennas showing both localized plasmon and surface lattice resonance modes. The ultrafast magnetization dynamics of the nanoantennas show a 3-fold resonant enhancement of the demagnetization efficiency. The degree of demagnetization is further tuned by activating surface lattice modes. This reveals a platform where ultrafast demagnetization is localized at the nanoscale and its extent can be controlled at will, rendering it multistate and potentially opening up so-far-unforeseen nanomagnetic neuromorphic-like systems operating at femtosecond time scales controlled by light.

With the exponentially increasing amount of digital cloud-stored information, the requirement for faster, denser, and energy-efficient alternatives to conventional magnetic memory storage poses one of the most important technological challenges of present times. Research on materials and devices that can meet these requirements spans various fields such as magneto-optics, ultrafast dynamics, spintronics, and more recently plasmonics.

A promising possibility for ultrafast switching of magnetization at the picosecond time scale opened up with the discovery of all-optical switching (AOS) of magnetization^1^ in the rare-earth transition-metal (RE-TM) alloy GdFeCo, wherein the magnetization could be reversed without an external magnetic field, using just ultrashort laser pulses. The phenomenon has since been observed in several other magnetic materials.^2−5^ Studies investigating the mechanism behind single-shot helicity-independent AOS reveal that a critical driving step of the process is fast and efficient demagnetization.^6−9^

For the technological viability of AOS, a crucial aspect is downscaling the bit size toward the nanoscale. While this has previously been attempted through nanopatterning,^10,11^ a potentially more energy-efficient avenue involves the use of nanoplasmonics.^12^ It has been experimentally demonstrated^13,14^ that nanoplasmons can confine and strongly enhance the electromagnetic field of light down toward the nanoscale, lowering the threshold fluence required for AOS and improving its energy efficiency. These observations motivate further exploration of the temporal evolution of nanoplasmon-assisted magnetization dynamics. Time-resolved magnetization dynamics of plasmon-based systems have previously been reported for self-assembled nanostructures^15^ and in continuous Co/Pt films,^16^ for both of which plasmon-driven enhancement of the demagnetization amplitude was observed. Here we explore plasmon-enhanced demagnetization in a material highly pertinent to AOS, namely the RE-TM alloy Tb_18_Co_82_. Tb-containing systems are prime candidates for dense magnetic memory storage due to their high perpendicular anisotropy and small stable-domain sizes arising from the strong spin–orbit coupling in Tb. The recent demonstration of single-shot helicity-independent magnetic switching in Tb/Co multilayers^17^ and TbGdCo alloys^18^ moves us closer to the realization of Tb-based magnetic memory architectures, making it critical to explore the combination of Tb-based magnetic nanomaterials with plasmon nanoantennas.^19^

We pinpoint plasmonic control of the demagnetization dynamics of an ordered rectangular array of truncated-nanocone Au antennas topped with ferrimagnetic Tb_18_Co_82_ alloy nanodisks. Crucially, in addition to the localized plasmon resonances in individual nanocone antennas,^20^ building up a rectangular-array magnetophotonic surface crystal introduces far-field diffractive coupling between the nanoantennas: i.e., surface lattice resonance modes.^21−23^ These modes can lead to a significant enhancement of the optical and magneto-optical response and sharper spectral features with a superior quality factor.^21−25^ Despite the extremely small amount of magnetic material in our system, we find a sizable pump–probe signal, which we attribute to the localized plasmon-enhanced magneto-optics.^26^ Upon exciting the plasmon resonance, the efficiency of demagnetization is strongly enhanced: more than 3 times stronger demagnetization is observed for on-resonance optical excitation compared to off-resonance pumping. Even more strikingly, the light coupling to the surface lattice resonance effectively modulates the demagnetization in the nanoferrimagnets on the antennas. Thus, it can act as a tuning “knob” controlling the efficiency of demagnetization and realizing an externally controlled multistate system.

The magnetophotonic surface crystal is built as a rectangular array of truncated-cone-shaped nanoantennas composed of an Au body topped with a thin TbCo nanodisk (Figure 1a,b; see Materials and Methods in the Supporting Information for nanofabrication details) mounted on a glass substrate. The magnetic anisotropy of the TbCo nanodisk is out-of-plane. Optical transmission of the array in the visible spectral range shows clear signatures of the localized surface plasmons of individual nanoantennas, complemented by the surface lattice mode due to the array structure (Figure 1c,d). In ordered arrays of nanoparticles, abrupt, discontinuous changes, known as Rayleigh anomalies, are seen in the optical spectra at certain wavelengths, depending on the array period and angle of incidence. These sharp changes occur for wavelengths at which one of the diffracted orders becomes parallel to the substrate (grazing incidence).^27−29^ The interference between the localized plasmon and a Rayleigh anomaly gives rise to surface lattice modes, which exhibit an angular dispersion similar to the Rayleigh anomaly. For the surface crystal studied here, the transmission spectra for a normally incident probe are similar along both orientations (Figure 1c,d): a single dip is observed corresponding to the localized plasmon resonance mode. However, the position of the transmission minimum shifts, going from ∼720 nm in the d_S+L_ direction to ∼760 nm in the d_L_ direction, likely arising from a small anisotropy between the orthogonal orientations introduced during fabrication. However, while probing along the d_S+L_ direction (Figure 1c), angular dispersion is observed in the transmission spectra, arising from the spectral overlap between the localized plasmon resonance and the Rayleigh anomaly in this direction. The transmission minimum observed at normal incidence shifts to longer wavelengths with increasing angle of incidence, and additional features are observed in the spectrum, due to the excitation of angularly dispersed surface lattice resonance modes. Such features are not observed in transmission spectra measured along the d_L_ orientation, where the single peak corresponding to the localized plasmon mode at ∼760 nm is observed for all angles of incidence. This is due to the spectral separation of the nanoantenna plasmon and the Rayleigh anomaly along this direction so that no surface lattice modes are observed (Figure 1d). The static optical and magneto-optical characterization of this system has been discussed in further detail in our previous work.^29^ Such a magnetophotonic surface crystal exhibits a very significant magneto-optical rotation in the spectral range of the nanoantenna plasmon, making it possible to study its static magneto-optical properties as well as magnetization dynamics.

**Figure 1:**
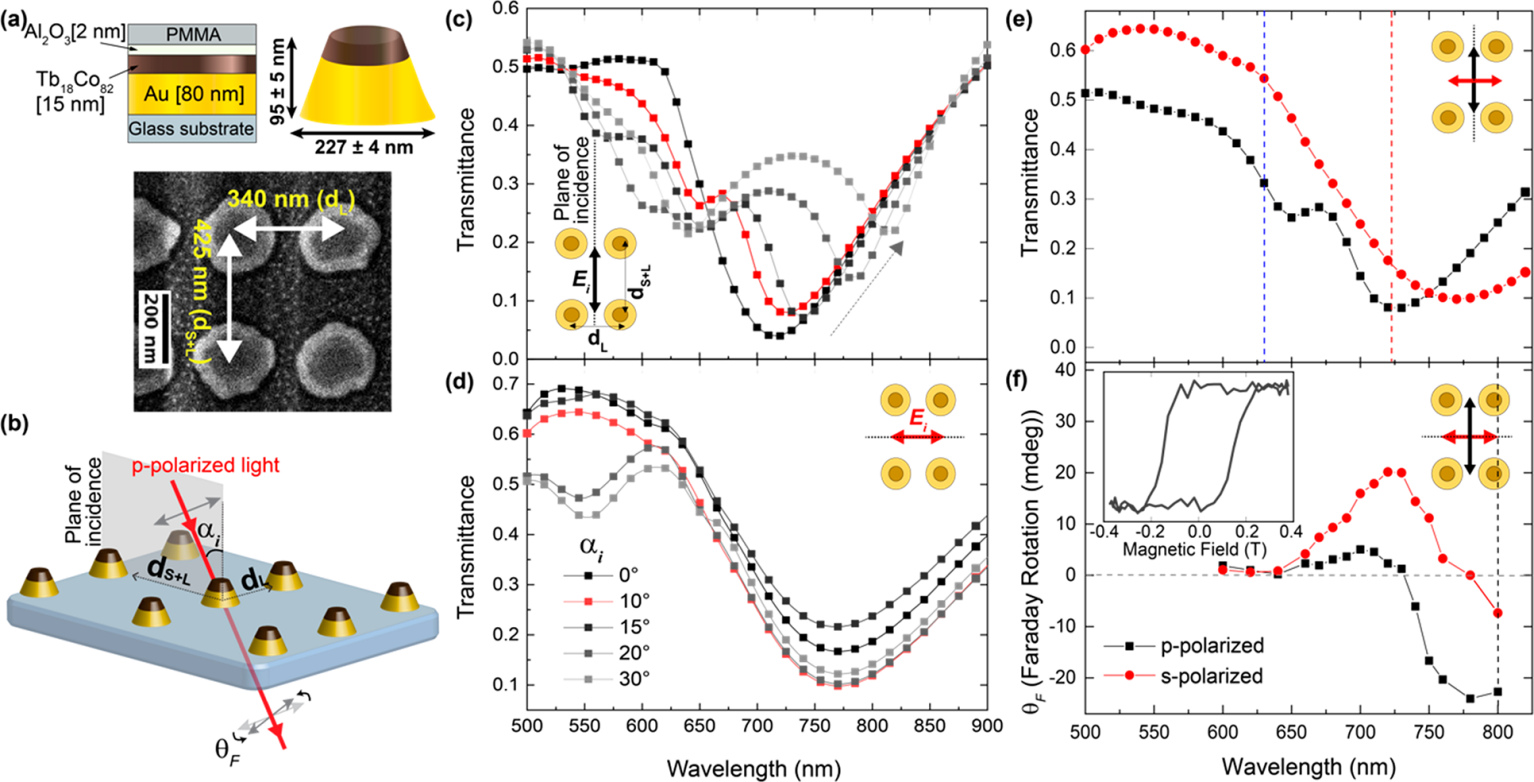
(a) (top) Schematic of the nanoantenna structure and composition and (bottom) SEM of the magnetophotonic surface crystal with two periodicities along the perpendicular directions labeled d_*S*+L_ and d_L_. (b) Experimental geometry for optical transmittance and magneto-optical spectra measurements indicating the plane of incidence and angle of incidence (α_i_) relative to the surface normal. (c) Optical transmittance for p-polarized incident light along the d_S+L_ direction, showing angular dispersion resulting from the interference of the localized plasmon and a surface lattice mode. (d) Optical transmittance for p-polarized light along the d_L_ direction with no angular dispersion. (e) Comparison of the optical transmittance (p-polarized incident light) along the two surface crystal directions for an angle of incidence of 10°. The pump wavelengths used in the pump–probe experiment are shown with dashed lines for off-resonance (630 nm, blue) and on-resonance (720 nm, red). (f) Polar Faraday rotation spectra (normal incidence) with s-polarized incident light along the d_L_ direction (red circles) and with p-polarized incident radiation along the d_S+L_ direction (black squares). The inset shows a normalized hysteresis loop for the nanocone antennas with a coercive field of 150 mT.

Figure 1e,f shows the transmission and magneto-optical spectra in the pump–probe experimental configuration—i.e. the magneto-optical spectrum for normal incidence (probe geometry) and the transmission spectra for 10° angle of incidence (pump geometry). The transmission spectra along d_L_ show a clear resonant peak corresponding to the nanoantennas’ plasmon close to the wavelength 760 nm. For spectra along d_S+L_, this peak is shifted to a wavelength of 720 nm, and additional sharper features corresponding to the Rayleigh anomaly and the resulting surface lattice resonance are observed. Simulations performed in our previous study^29^ reveal that, for 10° incidence, the [−1,0] Rayleigh anomaly, which strongly overlaps with the local plasmon mode, occurs at a wavelength of 690 nm. Thus, using an excitation wavelength of 720 nm, the nanoantennas’ localized plasmon resonance can be strongly excited along both directions, while along the d_S+L_ direction we are also close to the excitation wavelength of the surface lattice resonance. Note that the spectral position of the localized plasmon and the surface lattice resonances along both directions are not identical, and hence we chose the wavelength and geometry for which we can couple to the chosen resonances as strongly as possible using a single pump wavelength, to keep all other factors constant. The excitation at a wavelength of 630 nm, meanwhile, is far from the local plasmon resonances for both surface crystal directions. It is also located away from the [−1,0] surface lattice resonance but close to another feature, which likely corresponds to the Rayleigh anomaly [0,–1].^29^ We therefore refer to the two excitation wavelengths as on-resonance (720 nm) and off-resonance (630 nm), respectively, corresponding to the on- or off-resonant excitation of the localized plasmon mode. From the magneto-optical spectra, we find that, at the probe wavelength of 800 nm, the plasmon-enhanced magneto-optical signal for both directions is sufficiently high to probe the magnetization dynamics, for which the amplitude of transients is typically much lower than the total static magneto-optical response.

The inset in Figure 1f depicts a hysteresis loop measured as an averaged response over several nanocone antennas. Crucially, even confined to a nanoscale disk, TbCo retains its perpendicular magnetic anisotropy and has a substantial coercive field of 150 mT. This is close to the value of 200 mT observed for thin films of the same composition,^30^ indicating that it is indeed an excellent candidate for a nanosized magnetic memory. Comparing the magnetic properties of our system with an array of 200 nm diameter GdCo nanodots studied in ref (11), the TbCo nanomagnets here show nearly 3 times the coercive field despite having half of the diameter.

With the exceptions of plasmon nanoantenna-assisted single-shot helicity-independent switching in TbFeCo^13^ and the recent observation of helicity-independent toggle-switching in TbGdCo,^18^ Tb-TM alloys have been found to behave differently from Gd-TM alloys, in that they exhibit only multishot helicity-dependent switching,^30,31^ which is further restricted to alloys having a compensation temperature above room temperature.^2,30^ The compensation temperature of the Tb_18_Co_82_ composition that makes up the nanodisks in the surface crystal has been reported earlier by us to be below room temperature, and thus it displays pure thermal demagnetization, rather than AOS.^30^ However, the demagnetization dynamics of a multisublattice high-anisotropy magnetic system at the nanoscale in the presence of plasmon and lattice resonances could give us useful insights into understanding AOS behavior when observed in a similar system with a different Tb composition.

Figure 2 shows pump–probe dynamics for the two orthogonal directions of the magnetophotonic surface crystal (d_S+L_ and d_L_) and for on- and off-resonance pumping. The magnetization and transmission dynamics for the nanoantenna array are qualitatively similar, irrespective of the excitation wavelength or the direction of the surface crystal. For all cases, a femtosecond pump causes demagnetization of the surface crystal within 1 ps, followed by substantial magnetization recovery within ∼12 ps. Since the magnetic signal originates solely from the Tb_18_Co_82_, constituting a very small volume fraction of each nanoantenna, its amplitude is small with a rather poor signal-to-noise ratio.

**Figure 2:**
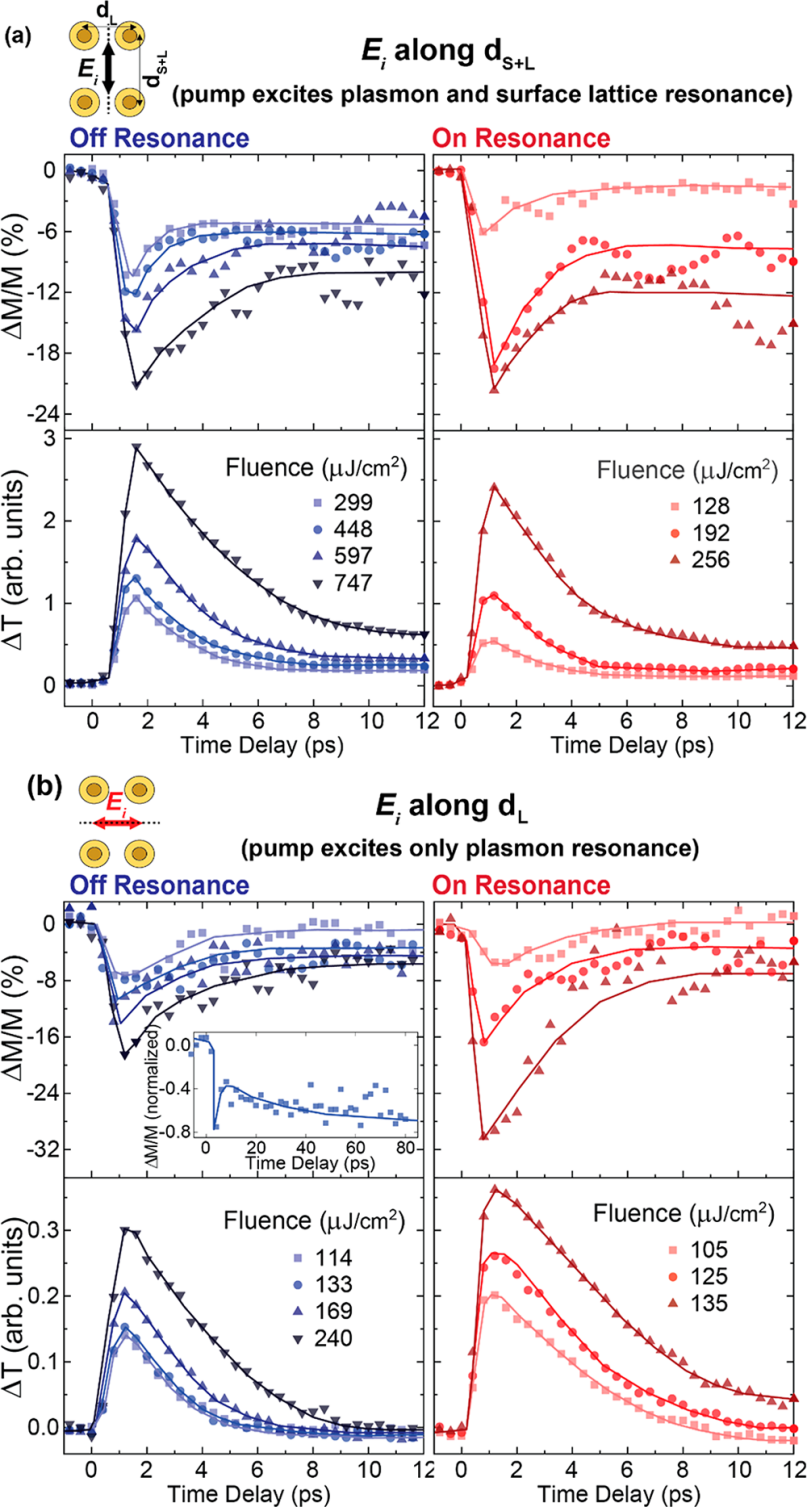
Magnetization (top) and transmission (bottom) dynamics for the magnetophotonic surface crystal across a time scale of 12 ps for different values of applied fluence, with probe polarization along d_*S*+L_ (a) and d_L_ (b) with off-resonance pumping (left) and on-resonance pumping (right) of the localized plasmon mode. The inset in the panel for off-resonant pumping in (b) shows the magnetization response over a longer time scale of 80 ps. Solid lines are a guide to the eye.

Quantitatively, however, the effects of resonant and off-resonant pumping of the localized plasmon mode are markedly different. The peak values of demagnetization are plotted in Figure 3 as a function of the applied fluence. In all cases, increasing the fluence results in increased demagnetization, as expected for heat-driven dynamics. The temperature-dependent magnetization *M*(*T*) in ferromagnetic materials is dictated by the Curie law , where *M*(0 K) is the magnetization at 0 K and *T*_Curie_ is the Curie temperature characteristic of the magnetic material. If heated beyond its Curie temperature, the material is completely demagnetized, since long-range magnetic correlations are lost. The pump-induced temperature rise in our system can be considered as linearly proportional to the incident fluence, so that we can use the Curie law to fit the fluence dependence of the degree of demagnetization for the data points shown in Figure 3 (note here that we fit Δ*M*/*M* (%) as a function of the applied fluence rather than the magnetization *M*). Such a fitting is shown in the same figure using the dashed lines for pumping along d_L_ and the solid lines for pumping along d_S+L_. The curves show a reasonable agreement with the observed behavior, indicating that the nanoantennas are heated close to the Curie temperature upon pump excitation. An approximate Curie temperature of 470 K is obtained from the fits.

**Figure 3:**
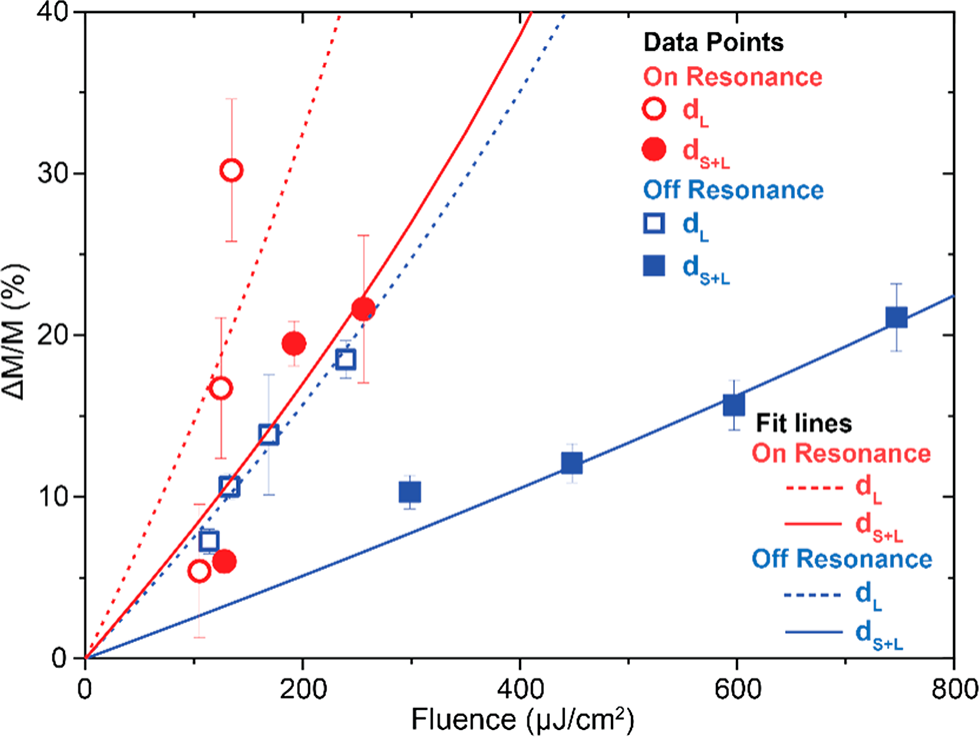
Peak values of demagnetization plotted as a function of applied fluence. The solid data points are for measurement along the d_S+L_ direction, and the open data points for measurement along the d_L_ direction. Solid lines are Curie-law fits for data points along dthe _S+L_ direction, and dashed lines are Curie-law fits for data points along the d_L_ direction. Red data points/lines denote on-resonance pumping and blue data points/lines off-resonance pumping of the localized plasmon mode.

Comparing the demagnetization for on- and off- resonance pumping, we find that for both surface crystal directions, on-resonance pumping of the localized plasmon mode leads to an enhancement of the demagnetization amplitude relative to off-resonance pumping. In fact, at the highest measured resonant-pump fluences, the demagnetization amplitude is more than 3 times as large as the amplitude for off-resonance pumping. This 3-fold enhancement in the demagnetization efficiency in these magnetophotonic nanoantennas has very promising implications for lowering the fluence requirement for all-optical magnetization switching, thereby improving its energy efficiency, in similar materials that do exhibit AOS.

More strikingly, comparing the demagnetization for excitation along the two orthogonal directions (d_S+L_ and d_L_), we find that for a given fluence *smaller* demagnetization is obtained when the localized plasmon resonance and surface lattice resonance are simultaneously engaged, as compared to exciting only the nanoantennas’ localized plasmons. We attribute this to the fact that, along d_S+L_, the surface lattice resonance delocalizes the intensity across the magnetophotonic surface crystal, whereas the localized plasmon resonance instead focuses the incident energy exclusively within the nanoantenna and the ferrimagnet. For deeper insight, we simulated the heating effect of the pumping on the nanocones. Figure 4a shows temperature maps for an individual nanocone antenna in the array for four different excitation conditions (on- and off- resonance, along both the d_L_ and d_S+L_ surface crystal directions) following excitation by a pump pulse with a fluence of 200 μJ/cm^2^. As shown in Figure 4b, the temperature profiles for these excitation conditions have been extracted for the nanoantenna corresponding to the nanocone base and to the top of the TbCo nanodisk (as indicated by the dashed lines in Figure 4a). At the nanocone base, for off-resonance excitation along the d_S+L_ direction, a significant asymmetry is observed in the temperature profile, which relates to the nonzero (10°) incidence angle of the laser beam. Note that this asymmetry is less pronounced for off-resonance excitation along the d_L_ direction: i.e., along the direction where the localized plasmon and the surface lattice resonance are spectrally distinct. For on-resonance excitation, however, the heating profile at the nanocone base is symmetrical for both surface crystal directions. For better visualization of the (a)symmetry of the temperature profile, the same temperature maps can be viewed from the top of the array (Figure S1 in the Supporting Information). For the TbCo nanodisk, the larger pump-induced heating for on-resonance excitation compared to off-resonance excitation is evident. However, simultaneous excitation of both the surface lattice resonance and the localized plasmon of the nanoantennas leads to a smaller temperature rise compared to excitation of the localized plasmon alone. The reduced heating of the TbCo nanodisk obtained for excitation along d_S+L_ agrees with our experimental observations of lower demagnetization along this surface crystal direction. This implies that the excitation of the surface lattice resonances in such arrays enables one to fine-tune the eventual degree of demagnetization in the nanoferrimagnets by diverting the incident fluence from the individual nanoantennas and redistributing it throughout the magnetophotonic surface crystal. We observe that, for off-resonance excitation, the simulated temperature profiles show a much smaller difference along the two orthogonal directions compared to the experimentally observed demagnetization. This could possibly arise from additional anisotropy introduced in the nanocones during the fabrication process.

**Figure 4:**
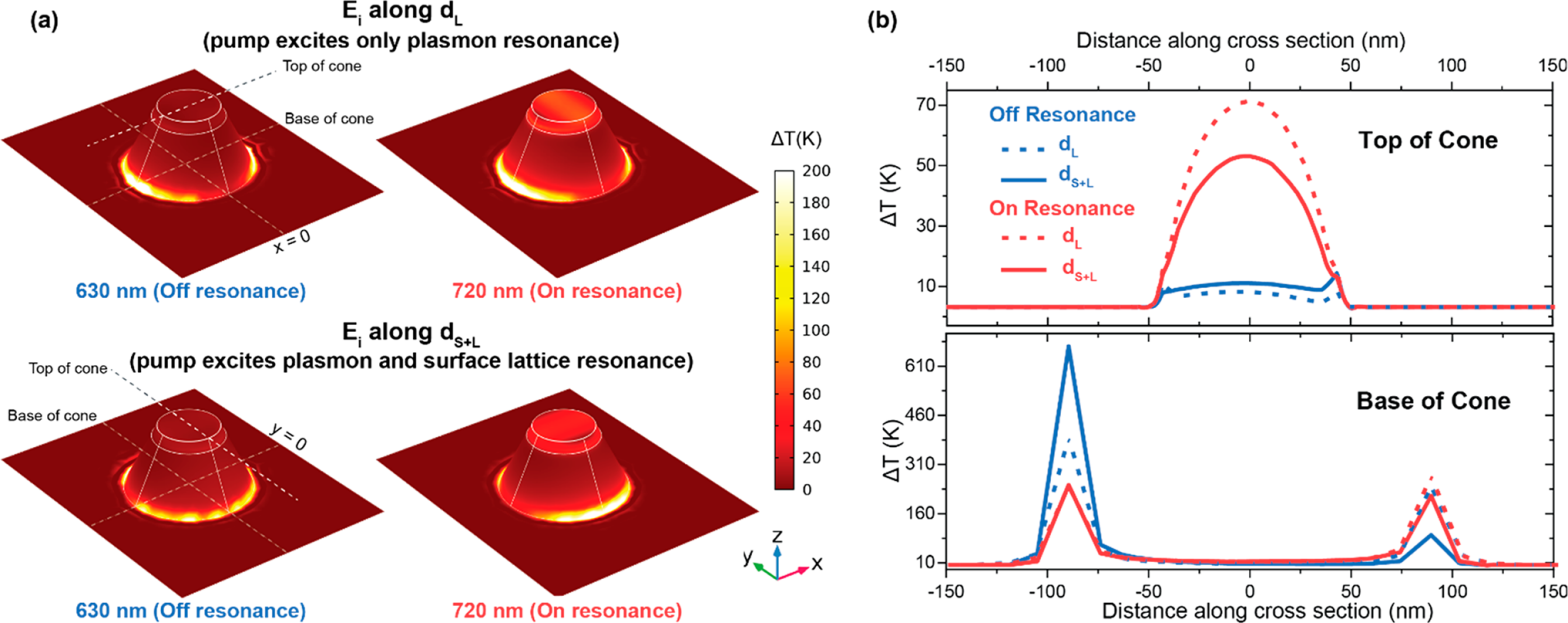
(a) Simulated temperature maps for a representative nanoantenna in the magnetophotonic surface crystal, corresponding to different excitation conditions: (top left) off-resonance along d_L_, (top right) on-resonance along d_L_, (bottom left) off-resonance along d_S+L_, and (bottom right) on resonance along d_S+L_, for an excitation fluence of 200 μJ/cm^2^. (b) Temperature profile of an individual nanoantenna, extracted from (top) the top of the TbCo nanodisk and (bottom) the base of the Au nanocone for the different excitation conditions. Red curves indicate on-resonance excitation, and blue curves indicate off-resonance excitation. Dashed curves indicate excitation along the d_L_ direction, and solid curves indicate excitation along d_S+L_ direction. The positions for which the temperature cuts have been obtained are indicated by the dashed lines in the subplots in (a). Note that the terms on- and off-resonance excitation correspond to the resonant/off-resonant excitation of the localized surface plasmon mode.

The qualitative similarity of the on- and off-resonance excitations is also supported by the long-time-scale magnetization dynamics observed for this system. A representative trace is shown in the inset in Figure 2 for off-resonant excitation along d_L_ (i.e, only pumping the localized plasmon). The response is normalized between 0 and 1 to show the qualitative behavior. According to ref (32), the observed demagnetization for all excitation conditions is of type II: an initial ultrafast demagnetization, followed by a partial relaxation and eventually a much slower demagnetization. In thin films of GdCo amorphous alloy,^33^ such behavior was attributed to distinct dynamics of the two magnetic sublattices, and the same reasoning can also be extended to TbCo alloys. The independence of the qualitative demagnetization dynamics from the surface crystal lattice direction and pumping wavelength at all measured time scales implies that neither resonance qualitatively affects the dynamics. However, the inherent time scales of the localized plasmon excitation and dephasing are on the order of a few femtoseconds^34−38^ and cannot be resolved by our 200 fs long probe. While such ultrafast processes have been well characterized and understood in the realms of nanophotonics and plasmonics, our findings hint strongly toward the potential of harvesting these properties in the field of magnetism.^39^

Note that the transmission dynamics have not been discussed in detail here. This is because, in plasmonic systems, resonant effects manifest in the absorption and scattering, with transmission often giving an incomplete picture of these effects. Demagnetization, on the other hand, is a direct consequence of fluence absorption in the system, and therefore changes resulting from excitation of resonances in the system can be visualized more accurately in the demagnetization dynamics.

Our results thus unequivocally establish the nanoscale magnetic properties and dynamics of ferrimagnetic TbCo nanodots coupled to plasmonic structures, as well as the enhancement of the demagnetization efficiency in these nanostructures due to the localized nanoantenna plasmon resonances, pointing toward their potential to realize energy efficiency in magnetic memory writing technology. However, the implications of exciting the surface lattice resonance are not as simple. On the one hand, it allows us to tune the demagnetization efficiency. On the other hand, its tendency to spread the intensity across the surface crystal lattice creates the possibility of constructive scattering interference between individual nanoantennas in the array.^40^ While avoiding coupling to the surface lattice mode with the normal incidence optical excitation, simply shrinking the lattice pitch might still present significant challenges in attaining the ultimate memory density of 1Tb/inch^2^ and beyond. The magnetic elements then should be on the order of a few nanometers, separated by a couple of tens of nanometers. This might result in reaching the superparamagnetic limit and the onset of near-field coupling of the plasmon antennas even at normal incidence excitation, rendering the demagnetization and eventual switching a result of a complex interplay of the far-field excitation and the near-field coupling. This might limit the minimum pitch size of the array and hence the maximum bit density that can be achieved for memory storage technology based on such ordered nanomagnetic arrays. It has been proposed that surface lattice resonances can even extend across multiple unit cells of nanoantenna arrays.^23^ Other simulations of electric near-field distributions in various 2D nanoparticle arrays^41−43^ find that the excitation of surface lattice resonance creates a drastic near-field enhancement *between* individual nanoantennas, whereas the excitation of plasmon resonance creates near-field enhancement confined to the edges of individual nanoantennas. This intensity-spreading effect could also affect the relaxation dynamics of the nanoantennas, which could not be presently studied in depth for our system due to the small magnetic signal. This highlights a need to explore the role of surface lattice resonances in the dynamics of magnetization and switching processes in ordered magnetophotonic arrays in greater detail.

Despite the ambiguity in the effects of utilizing surface lattice resonances for dense memory architectures based in individual nanodots as bits, the fine-tuning of the demagnetization amplitude by the collective lattice resonance opens up an alternative functionality at the level of the entire surface crystal. Engaging the lattice of the surface crystal, we can potentially obtain picosecond-lived multilevel magnetization states in such magnetoplasmonic system, primarily controlled by pumping along the two distinct crystal directions and possibly by adjusting the angle of incidence along d_S+L_. The multilevel “memory” signature upon femtosecond-pulse excitation could be applied as a “synaptic weight” in terms of neuromorphic computations^44,45^ in the ultrafast magneto-optical response of the system, tunable by the mutual coupling between nanoantennas through scattered electromagnetic fields, in turn modulated by the pump wavelength, angle of incidence, and lattice geometry. Furthermore, the long-range electromagnetic coupling facilitated by the surface lattice resonances could yield “synapses” between multiple, if not all, nanoantennas of the magnetophotonic surface crystal and neighboring surfaces comprising stacks of these, as was done for the case of diffractive deep neural network architectures (D^2^NN) by Lin et al.^46^ Another potentially useful aspect is the possibility of engaging a nonlinear activation function, originating from the scattering and plasmonic characteristics of the individual hybrid magnetophotonic nanoantennas, ultimately defined by the size, shape, and materials used.^47^ These are key required ingredients, which are not readily available in the current experimental D^2^NN approaches,^48,49^ to realize hardware versions of neuromorphic computing architectures based on electromagnetic waves, thereby promising energy-efficient, high-speed parallel computing. In this context the ferrimagnetic bit size is not a relevant performance indicator, as the entire surface crystal would perform the neuromorphic function by engaging the localized and surface lattice modes. We envision such femtosecond-activated magnetophotonic crystals to eventually become a functional part in photonic neuromorphic systems.^50^

To summarize, we present the ultrafast demagnetization dynamics in a magnetophotonic surface crystal composed of Au-Tb_18_Co_82_ truncated-nanocone antennas. The system is pumped on- and off-resonance to excite the localized nanoantenna plasmons and surface lattice resonance modes to study their effect on the dynamics. While the qualitative responses of the crystal to on- and off-resonant excitation are similar, clear quantitative differences are revealed. We observe substantial nanoplasmon-induced enhancement of demagnetization in individual nanoantennas, where the demagnetization efficiency increases up to 3 times for on-resonant pumping compared to off-resonant pumping. Conversely, the surface lattice resonance mode tunes the degree of demagnetization by redistributing the scattering intensity and nanoscale heat transfer throughout the crystal. This creates a finely controlled nanosystem featuring a multilevel demagnetization on demand. The plasmon-controlled enhancement of the demagnetization dynamics in nanoscale ferrimagnets is promising for the future optimization of such hybrid magnetophotonic materials to realize energy-efficient nanoscale memory architectures.

## Supporting Information Available

is available and contains. . The Supporting Information is available free of charge at https://pubs.acs.org/doi/10.1021/acs.nanolett.2c00769.

Sample fabrication procedure, description of optical and magneto-optical characterization, time-resolved magnetization dynamics experimental procedure, electromagnetic and thermal simulation procedure, and a figure giving thermal simulations (top view of temperature profile of a nanocone for different excitation conditions) (PDF)

## Abbreviations

AOS: all-optical switching
RE-TM: rare-earth transition metal
