# Supplementary material for: Ultrafast Demagnetization Control in Magnetophotonic Surface Crystals
Source: Nano Lett. 2022 Nov 2;22(23):9773–80. doi: 10.1021/acs.nanolett.2c00769 (PMC9756331; doi:10.1021/acs.nanolett.2c00769)
Supplement: Supplementary file 1 — nl2c00769_si_001.pdf [file nl2c00769_si_001.pdf]

Ultrafast Demagnetization Control in Magnetophotonic Surface Crystals

Kshiti Mishra,[1] Richard M. Rowan-Robinson,[2,†] Agne Ciuciulkaite,[2] Carl S. Davies,[1,3] Alexandre Dmitriev,[4] Vassilios Kapaklis,[2] Alexey V. Kimel,[1] and Andrei Kirilyuk*[1,3]*

[1]Radboud University, Institute for Molecules and Materials, Heyendaalseweg 135, 6525 AJ Nijmegen, The Netherlands

[2]Department of Physics and Astronomy, Uppsala University, Box 516, SE-75120 Uppsala, Sweden

[3]FELIX Laboratory, Radboud University, Toernooiveld 7, 6525 ED Nijmegen, The Netherlands

[4]Department of Physics, University of Gothenburg, SE-412 96 Göteborg, Sweden

† *(Present address: Department of Material Science and Engineering, University of Sheffield, Sheffield, S1 3JD, United Kingdom)*

KEYWORDS: Magnetoplasmonics; Magnetophotonics; Ultrafast Magnetization Dynamics; All-Optical Switching; Demagnetization; Surface Lattice Resonances

SUPPORTING INFORMATION

Materials and Methods:

Sample Fabrication

The magnetoplasmonic nanoantenna array was fabricated using the method outlined by Horrer *et al.* (46) An Au(80 nm) film was deposited using electron-beam evaporation onto glass substrates, on which Al_2O_3(3.5 nm)/$Tb_{18}Co_{82}$(15 nm)/Al_2O_3(2 nm) films were sputter deposited to obtain the layered structure: Au(80 nm)/Al_2O_3(3.5 nm)/$Tb_{18}Co_{82}$(15 nm)/Al_2O_3(2 nm). The $Tb_{18}Co_{82}$ alloy layer was deposited employing magnetron co-sputtering. The additional thin Al_2O_3 layers were used as capping and isolating layers for the $Tb_{18}Co_{82}$. The composition of the $Tb_{18}Co_{82}$ layer was confirmed using Rutherford back scattering and the layer thickness was determined by X-ray reflectivity measurements. Electron beam lithography was used to define

disk shaped apertures in a MicroChem 496PMMA A4 electron-beam resist, arranged in a rectangular lattice with the periodicities of d_{S+L} = 425 nm and d_L = 340 nm. An Al mask was then deposited using electron-beam evaporation, followed by removal of the PMMA mask with Acetone. The resulting structure was Ar-ion milled at a 5° incidence angle with sample rotation, removing all material unprotected by the Al mask. As a result, a conical profile is induced through a combination of the small lateral component of the milling which depends to some extent on the small milling incidence angle(47). Finally, any remaining Al mask was removed with selective etching using the photoresist developer Microdeposit 351. The motivation for the choice of the conical shape of the array elements is described in detail by Rowan-Robinson *et al.*(29).

Optical and Magneto-optical Characterization

Optical transmittance spectra were measured using a monochromator in combination with an Hg arc lamp white light source. Spectra were measured with incident light polarization parallel to both orthogonal directions of the rectangular arrays for various angles of incidence. Magneto-optical spectra in Faraday geometry were measured using the same source using a photoelastic modulation technique.

Time-resolved Magnetization Dynamics

An all-optical pump-probe setup was used to study the magnetization dynamics of the sample. Both pump and probe beams were derived from a 1 kHz Ti:Sapphire amplified laser system with a central wavelength of 800 nm and a pulse duration of 200 fs at the sample position. The pump wavelength was tuned by optical parametric amplification. The probe was at normal incidence to the sample surface, focused to a spot size of 125 μm, whereas the pump beam was incident at 10°. The pump wavelength was varied between resonant (720 nm) and off resonant (630 nm) conditions. The spot size for the pump at both wavelengths was adjusted to ~ 200 μm. Both pump and probe beams were horizontally (p-) polarized and the sample was rotated in order to probe along the two orthogonal directions. A constant saturating out-of-plane magnetic field was applied through the course of each measurement to reinitialize the magnetic state of the system to a consistent state at the beginning of each pump-probe event. Magnetization dynamics were measured for opposite magnetic field polarities and subtracted to remove any non-magnetic contributions.

Electromagnetic and Thermal Simulations

Three-dimensional electrodynamic calculations of the optical response and the temperature changes in our truncated-nanocone structures were performed solving Maxwell's equations and subsequentially heat diffusion equations, in a Finite Element Method (FEM) framework implemented in COMSOL Multiphysics[48]. Details of the electromagnetic simulations have been presented in depth in ref. [27]. The presented temperature profiles were determined by estimating the dissipated energy from the electromagnetic field during the duration of the laser pulse (200 fs) and subsequent calculation of the heat distribution accounting for the nanocone materials parameters to the best of our knowledge(29,30).

Thermal Simulations: Top view of temperature profile of nanocone for different excitation conditions:

Figure S1 shows the top view of the temperature profiles for a representative nanocone in the array, in response to pump excitation for four conditions: excitation on- and off- resonance, along d_L and d_{S+L}. The differences between pump-induced temperature changes for the two directions are clearly visible. At the base of the nanocone for off-resonant excitation, a pronounced asymmetry is observed in the temperature profile, especially along the d_{S+L} direction. For resonant excitation, the temperature profile at the base is more symmetric. At the top of the TbCo nanodisk, stronger heating for on-resonant excitation is evident compared to

off-resonant excitation, and the differences in heating for on-resonant excitation along the two directions (d_L and d_{S+L}) are also better visible.

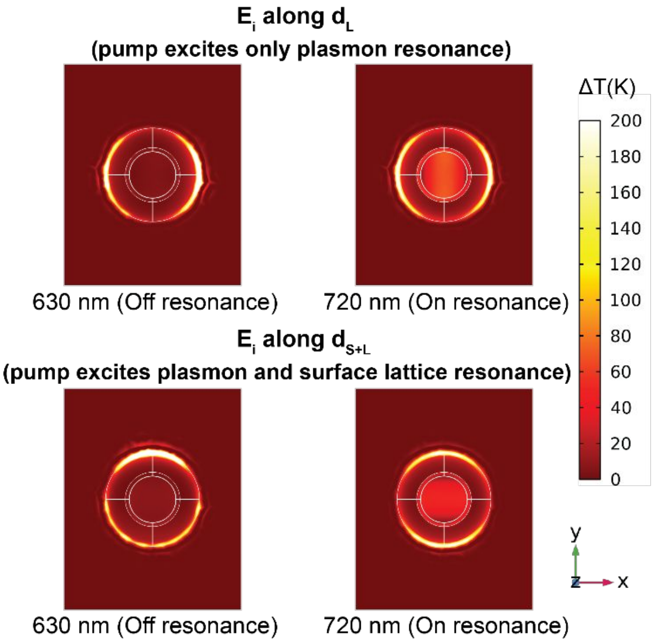

Figure S1. Electromagnetic simulations of temperature maps viewed from the top for a representative nanocone in the array, corresponding to different excitation conditions: (top, left panel) off resonance along d_L, (top, right panel) on resonance along d_L, (bottom, left panel) off resonance along d_{S+L}, (bottom, right panel) on resonance along d_{S+L}, for an excitation fluence of 200 μJ/cm^2.
